# The culture microenvironment of juvenile idiopathic arthritis synovial fibroblasts is favorable for endochondral bone formation through BMP4 and repressed by chondrocytes

**DOI:** 10.1186/s12969-021-00556-8

**Published:** 2021-05-12

**Authors:** Megan M. Simonds, Amanda R. Schlefman, Suzanne M. McCahan, Kathleen E. Sullivan, Carlos D. Rose, Anne Marie C. Brescia

**Affiliations:** 1 Nemours Biomedical Research, Nemours/Alfred I. duPont Hospital for Children, 1701 Rockland Rd, Wilmington, DE 19803 USA; 2 Rheumatology, Johns Hopkins All Children’s Hospital, St. Petersburg, FL USA; 3 Allergy and Immunology, Children’s Hospital of Philadelphia, Philadelphia, PA USA; 4 Division of Rheumatology, Nemours/Alfred I. duPont Hospital for Children, 1701 Rockland Rd, Wilmington, DE 19803 USA

**Keywords:** Fibroblast, Synoviocyte, Chondrocyte, TGFβ, BMP4, BMP antagonists, Hypertrophy, Growth disturbances, Endochondral bone formation, Juvenile idiopathic arthritis

## Abstract

**Background:**

We examined influences of conditioned media from chondrocytes (Ch) on juvenile idiopathic arthritis synovial fibroblasts (JFLS) and potential for JFLS to undergo endochondral bone formation (EBF).

**Methods:**

Primary cells from three control fibroblast-like synoviocytes (CFLS) and three JFLS were cultured in Ch-conditioned media and compared with untreated fibroblast-like synoviocytes (FLS). RNA was analyzed by ClariomS microarray. FLS cells cultured in conditioned media were exposed to either TGFBR1 inhibitor LY3200882 or exogenous BMP4 and compared with FLS cultured in conditioned media from Ch (JFLS-Ch). Media supernatants were analyzed by ELISA.

**Results:**

In culture, JFLS downregulate BMP2 and its receptor BMPR1a while upregulating BMP antagonists (NOG and CHRD) and express genes (MMP9, PCNA, MMP12) and proteins (COL2, COLX, COMP) associated with chondrocytes. Important TGFβ superfamily member gene expression (TGFBI, MMP9, COL1A1, SOX6, and MMP2) is downregulated when JFLS are cultured in Ch-conditioned media. COL2, COLX and COMP protein expression decreases in JFLS-Ch. BMP antagonist protein (NOG, CHRD, GREM, and FST) secretion is significantly increased in JFLS-Ch. Protein phosphorylation increases in JFLS-Ch exposed to exogenous BMP4, and chondrocyte-like phenotype is restored in BMP4 presence, evidenced by increased secretion of COL2 and COLX. Inhibition of TGFBR1 in JFLS-Ch results in overexpression of COL2.

**Conclusions:**

JFLS are chondrocyte-like, and Ch-conditioned media can abrogate this phenotype. The addition of exogenous BMP4 causes JFLS-Ch to restore this chondrocyte-like phenotype, suggesting that JFLS create a microenvironment favorable for endochondral bone formation, thereby contributing to joint growth disturbances in juvenile idiopathic arthritis.

**Supplementary Information:**

The online version contains supplementary material available at 10.1186/s12969-021-00556-8.

## Background

Juvenile idiopathic arthritis (JIA) is the most common rheumatic disease in children with an increased risk of joint destruction leading to disability in more severe subtypes [1, 2]. In particular, children suffering from JIA have accelerated bone growth in affected joints. While this bony overgrowth has been associated with increased production of proinflammatory cytokines, which may influence growth through a local effect in the growth plates of long bones, little is known about how cell types in the affected joint and growth-signaling pathways contribute to this abnormal bony overgrowth [3].

While we have shown previously that there is evidence that fibroblast-like synoviocytes (FLS) play a critical role as gatekeepers and mediators of inflammatory response in JIA [4], it is likely that these cells also play a role in joint growth disturbances seen in the progression of this disease. Here, we examine the interaction between JIA FLS (JFLS) and chondrocytes (Ch) because not only are Ch a prominent cell type in the joint, these cells are also widely accepted as playing a critical role in new bone growth through the process of endochondral bone formation (EBF) [5, 6].

During EBF, cartilage located at the growth plate of the long bone progresses from immature to mature and eventually provides the scaffolding for new bone to form. Specifically, Ch proliferate, hypertrophy, and undergo apoptosis, leaving behind a matrix for osteoblasts to invade [7, 8].

Bone morphogenetic proteins (BMPs) are part of the larger TGFβ superfamily. Ch produce many TGFβ and BMP ligands. During EBF, TGFβ causes Ch to proliferate but not mature, while BMPs drive Ch differentiation to hypertrophic chondrocytes (HCh) as evidenced by collagen X (COLX) and bone-derived alkaline phosphatase (ALP) expression [8].

In this study, we propose that JFLS transform into a phenotype capable of creating a microenvironment favorable for EBF. In addition to determining the influence normal Ch have on diseased FLS, we also examined intracellular signaling in the TGFβ/BMP pathway and how it regulates the HCh-like phenotype of JFLS in culture.

## Methods

### Selection of samples

Synovial fluid samples were obtained from our Institutional Review Board-approved repository. Patients who underwent clinically indicated arthrocentesis were offered inclusion into the repository, and informed consent was obtained. We selected primary cells from three subjects with persistent oligoarticular JIA, who had no prior steroid injections and were on nonsteroidal anti-inflammatory drugs (NSAIDs). We procured three normal primary human chondrocyte cell samples (402 K-05a) and three normal primary human FLS cell samples from Cell Applications, Inc. (408 K-05a). All purchased primary cells were from individual donors.

### FLS-conditioned media

To obtain conditioned media, three Ch samples were grown to confluence. Media was freshly replaced, and cells were incubated an additional 48 H*. media* from all three Ch samples were pooled and used as Ch-conditioned media. Three CFLS and three JFLS cell samples were grown until confluence then washed with PBS, trypsinized, and resuspended in the pooled Ch-conditioned media or normal FLS growth media as a control. Cell culture supernatants and cell pellet lysates were collected at 6 and 24 h after exposure to Ch-conditioned media.

### Inhibition of TGFβ

Three CFLS and three JFLS samples were grown until confluence then washed with PBS and trypsinized, and each sample was resuspended in the pooled Ch-conditioned media. After plating in 6-well plates, cells were incubated in Ch-conditioned media for 24 H*. media* was then aspirated, and Ch-conditioned media containing 100 mg of transforming growth factor-β receptor type 1 (TGFBR1) inhibitor LY3200882 (SelleckChem S8772) was added to each well. This was calculated using the datasheet provided by the manufacturer. Cell culture supernatants and cell pellet lysates were collected at 6 and 24 h after treatment.

### Addition of exogenous BMP4

Three CFLS and three JFLS samples were grown until confluence then washed with PBS and trypsinized, and each sample was resuspended in the pooled Ch-conditioned media. After plating in 6-well plates, cells were incubated in Ch-conditioned media for 24 H*. media* was then aspirated, and Ch-conditioned media containing 1000 ng/ml of BMP4 (R&D 314-BP) was added to each well [9]. Cell culture supernatants and cell pellet lysates were collected at 6 and 24 h after treatment.

### Enzyme-linked immunosorbent assay

Protein concentrations from cell culture supernatants were measured using Bradford assay. ELISA kits from LifeSpan Biosciences, Inc. were used to detect gremlin (LS-F21084), noggin (LS-F24239), collagen II (LS-F26824), and collagen X (LS-F13131). ELISA kits from Raybiotech Inc. were used to detect follistatin (ELH-FOLLISTATIN) and chordin (ELH-CHRDL). Alkaline Phosphatase Assay Kit was purchased from Abcam Inc. and performed according to the manufacturer’s protocol (ab83369).

### Phosphorylation antibody arrays

Human TGFβ Pathway Phosphorylation Arrays (AAH-TGFB-1) were performed on cell lysates according to the manufacturer’s protocol. Changes in phosphorylation were measured using ‘Integrated Density’ through ImageJ.

### GeneChip whole transcriptome expression analysis

Arrays were processed following the standard Affymetrix protocol [10]. Gene expression was determined using the SST-RMA algorithm in Expression Console (Affymetrix).

### Data analysis

LIMMA analysis was performed using the R package, limma [11], to determine differentially expressed genes between FLS cultured in different conditions with 1% FDR considered as significant. Gene expression of CFLS cultured for 6 h was compared with that of CFLS-Ch cultured for 6 h. Gene expression of JFLS cultured for 6 h was compare with that of JFLS-Ch cultured for 24 h. The 277 differentially expressed genes with a 1% FDR were input into Ingenuity Pathway Analysis and top pathways, networks, and analysis molecules were analyzed. From the IPA analysis we generated curated lists of genes of interest. Ratios comparing 24-h to 6-h time points were calculated using linear intensity for each sample for CFLS, JFLS, CFLS-Ch, and JFLS-Ch. Specifically, we used values created from RMA and performed an anti-log transformation to obtain the linear intensity. Each linear intensity value was normalized to GAPDH. Fold changes were calculated by dividing intensity values at 24-h from values at 6-h. Using excel, we calculated the log value for each of these ratios to distinguish whether gene expression was increasing or decreasing. Changes over time were graphed, standard deviation and t-tests were calculated using Excel. We used t-tests to determine statistical significance.

For all ELISA, each biological replicate was plated in triplicate. Optical density readings were converted to pg/ml using standard curves. Total protein measurements were plotted after 24 h in culture, and t-tests were used to determine statistical differences between two groups.

For phosphorylation antibody arrays, the integrated density of each dot was measured by outlining it and using the Analyze/Measure command in ImageJ. After background correction, profile plots were obtained for each row of dots (Analyze/Plot Profile). “Integrated Density” was enabled to create a circular selection that was dragged over the dots, and intensity was measured. Data were exported into Excel for calculations. The total intensity after 24 h in culture was used to determine differences in phosphorylation between two comparisons. A standard t-test was performed to determine significance.

## Results

### JFLS have decreased expression of genes specific to BMP signaling

**Fig. 1:**
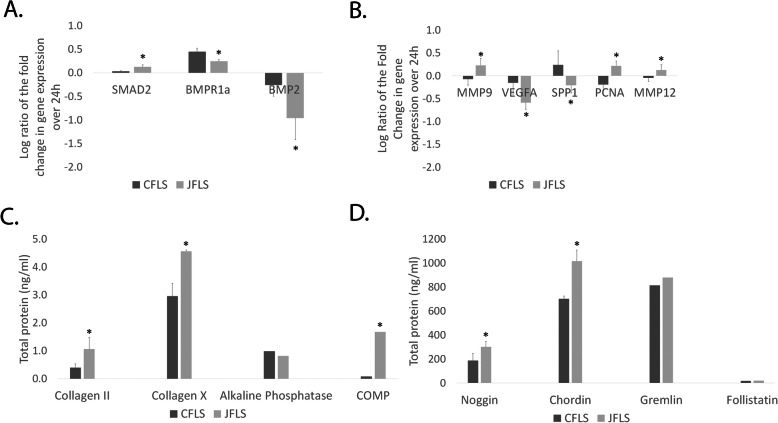
Changes in gene expression over 24 h between control FLS (CFLS) and JIA FLS (JFLS). JFLS favor TGFβ through increased expression of SMAD2 (*p* = 0.016) and downregulate BMP receptor BMPR1a (*p* = 0.007) and BMP ligand BMP2 (*p* = 0.041) compared with CFLS (**a**). JFLS have increased expression of MMP9 (*p* = 0.035), PCNA (*p* = 0.006), and MMP12 (*p* = 0.049), genes typically associated with prehypertrophic and hypertrophic chondrocytes (Ch) and decreased expression of VEGFA (*p* = 0.032) and SPP1 (*p* = 0.049), genes normally expressed by perichondral and endochondral osteoblasts during endochondral bone formation (EBF) (**b**). Total protein expression of Ch markers was measured on cell culture supernatants using ELISA. JFLS secrete significantly more collagen II (COL2 *p* = 0.029), collagen X (COLX *p* = 0.026), and cartilage oligomeric matrix protein (COMP *p* = 0.023) when compared with CFLS (**c**). Total protein expression of BMP antagonists was measured on cell culture supernatants using ELISA. JFLS overexpress chordin (*p* = 0.020) and noggin (*p* = 0.030) compared with CFLS (**d**)

Based on our microarray analysis discussed later in this manuscript and given that TGFβ and BMP contribute to the pathogenesis of rheumatoid arthritis (RA), and TGFβ/BMP generally regulate endochondral bone formation [9, 12–14], we manually curated a list of 27 genes related to TGFβ/BMP signaling including Smads, BMP ligands and receptors, and TGFβ ligands and receptors to examine this prominent signaling pathway in JFLS using Ingenuity Pathway Analysis (IPA). Gene expression was determined by plotting the log ratio of the fold change in linear expression over 24 h in cells cultured in regular media. Elevated SMAD2 (*p* = 0.016) gene expression demonstrates upregulated TGFβ gene expression in JFLS compared with CFLS. JFLS downregulate receptor and ligand gene expression specific to BMP signaling compared with CFLS (BMPR1a, *p* = 0.007) and BMP2, *p* = 0.041) (Fig. 1a).

### JFLS differentiate along a chondrocyte-like lineage

We previously reported that JFLS have a chondrocyte-like phenotype [9]. On the basis of our aforementioned findings, using IPA, we examined a second curated list of 47 genes involved in cell maturation and differentiation. Consequently, we explored genes specifically related to chondrocyte differentiation in relation to endochondral bone formation. Changes in gene expression were determined by calculating the log ratio of the fold change of linear expression over 24 h. Genes expressed by prehypertrophic and hypertrophic chondrocytes are overexpressed in JFLS (MMP9 *p* = 0.035, PCNA *p* = 0.006, MMP12 *p* = 0.049) compared with CFLS, while genes typically expressed by perichondral and endochondral osteoblasts during EBF have decreased gene expression in JFLS compared with CFLS (VEGFA *p* = 0.032 and SPP1 *p* = 0.049) (Fig. 1b) [15–19]. TGFβ signaling plays a fundamental role during EBF [20]. Increased gene expression of SMAD2 coupled with expression of genes related to HCh suggest JFLS gene expression favors differentiation toward chondrocyte lineage through TGFβ signaling.

### Downstream protein expression supports a prehypertrophic/hypertrophic chondrocyte-like phenotype in JFLS

Since our gene expression results suggest that JFLS simulate mature cartilage, we measured total protein amounts of downstream proteins of well-characterized markers of hypertrophic chondrocytes using ELISA. JFLS significantly overexpress COL2 (*p* = 0.029), COLX (*p* = 0.026), and COMP (*p* = 0.023), common protein markers expressed by mature and hypertrophic chondrocytes, compared with untreated CFLS (Fig. 1c). There were no significant differences between JFLS and CFLS when examining protein expression of bone-derived ALP, a novel marker of disease progression in RA (Fig. 1c) [21].

### JFLS favor BMP antagonists with a high affinity for BMP4

Given that BMP can promote long bone growth through EBF and the downregulation of BMP-specific genes in JFLS, we used ELISA to measure total protein expression levels of prominent BMP antagonists. JFLS have significantly increased protein expression of noggin (NOG) (*p* = 0.030) and chordin (CHRD) (*p* = 0.020) compared with CFLS, while gremlin (GREM) and follistatin (FST) remained unchanged between the two cell types, suggesting that JFLS regulate BMP signaling through protein antagonists with a high affinity for BMP4 specifically (Fig. 1d) [22].

### Gene expression of CFLS and JFLS is altered when cultured in Ch-conditioned media

**Fig. 2:**
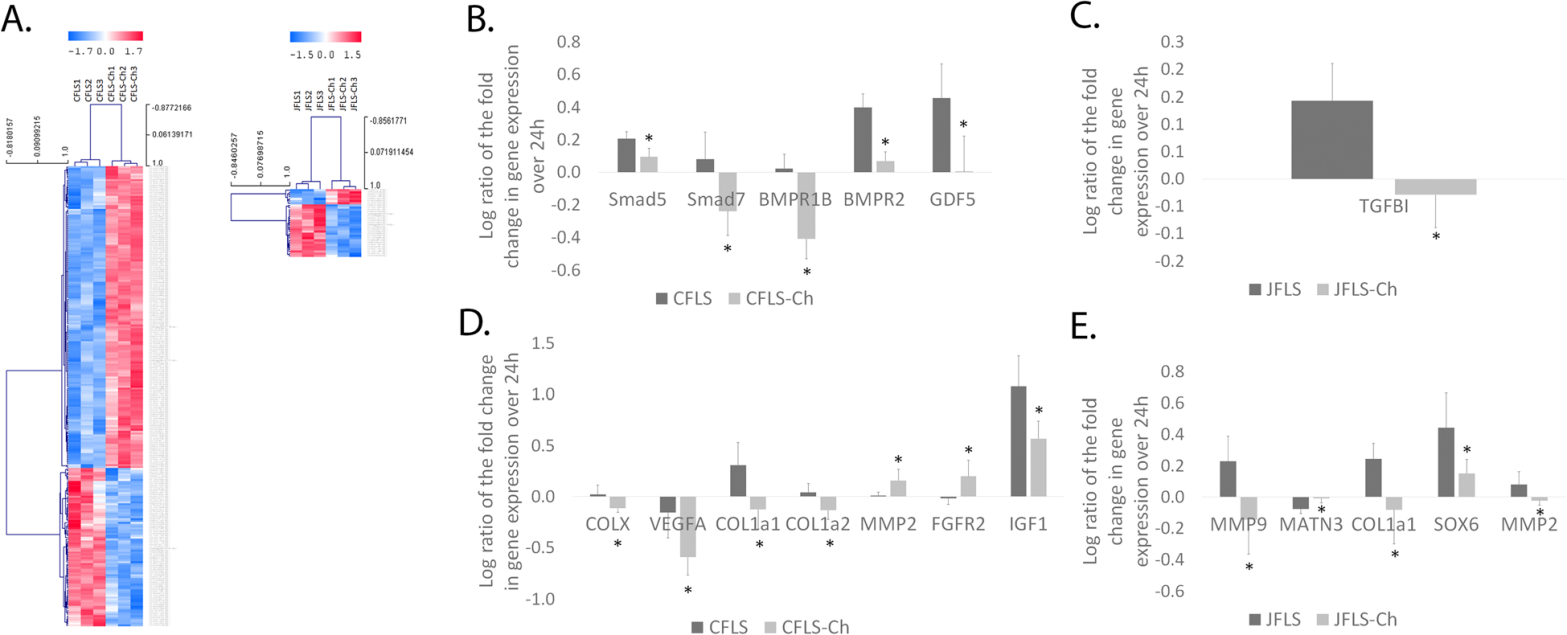
Unsupervised hierarchal clustering comparing FLS and FLS cultured in conditioned media from Ch after 6 h in culture and differentially expressed genes in these cells. There are 247 differentially expressed genes in CFLS vs CFLS cultured in conditioned media from chondrocytes (CFLS-Ch) and 31 genes are differentially expressed in JFLS vs conditioned media from chondrocytes (JFLS-Ch) (1% FDR) (**a**). Using a manually curated list of genes specific to TGFβ and BMP signaling pathways, we examined changes in gene expression over 24 h using linear expression values. CFLS-Ch have decreased expression of BMPR1b (*p* = 0.003), BMPR2 (*p* = 0.002), GDF5 (*p* = 0.030), SMAD5 (*p* = 0.023), and SMAD7 (*p* = 0.035) compared with CFLS (**b**). JFLS-Ch have decreased expression of TGFBI compared with JFLS (*p* = 0.016) (**c**). Using a manually curated list of genes specific to Ch differentiation and maturation, changes in the log ratio of the fold change of linear gene expression over 24 h revealed that CFLS-Ch downregulate COLX (*p* = 0.037), VEGFA (*p* = 0.034), COL1A1 (*p* = 0.031), and COL1A2 (*p* = 0.036) compared with CFLS (**d**). CFLS-Ch overexpress MMP2 (*p* = 0.048), FGFR2 (*p* = 0.050), and IGF1 (*p* = 0.027) compared with CFLS (**d**). JFLS-Ch have decreased expression of MMP9 (*p* = 0.038), COL1A1 (*p* = 0.042), SOX6 (*p* = 0.049), and MMP2 (*p* = 0.050) compared with untreated JFLS. JFLS-Ch upregulate expression of MATN3 (*p* = 0.023) when compared with JFLS (**e**)

It has been widely accepted that EBF relies upon the death of HCh to provide the scaffolding for new bone cells to invade [5]. Utilizing an unbiased approach to globally characterize FLS and FLS cultured in conditioned media from Ch, we discovered distinct discordances in gene expression. LIMMA analysis revealed 247 genes differentially expressed in CFLS vs CFLS cultured in conditioned media from chondrocytes (CFLS-Ch) and 31 genes differentially expressed in JFLS vs conditioned media from chondrocytes (JFLS-Ch) after 6 h in culture (1% FDR) (Supplemental Table 1). We generated separated unsupervised hierarchical clustering of CFLS compared with CFLS-Ch and JFLS compared with JFLS-Ch after 6 h in culture using these genes (Fig. 2a). Both CFLS and JFLS have gene expression patterns with divergent features when cells were cultured in Ch-conditioned media. Importing these transcriptome-wide significant genes into Ingenuity Pathway Analysis (IPA) revealed the ‘Top Ready-Analysis Molecules’ for each comparison (CFLS vs CFLS-Ch or JFLS vs JFLS-Ch). While these genes were not specifically from the 277 genes with a 1% FDR after LIMMA analysis, the genes can be readily associated with a biological processes or disease pathogenesis. In both comparisons, the top molecules regulate or are regulated by the TGFβ signaling pathway, MAPK signaling pathway, or inflammatory responses in cells. These genes also have features related to tissue development, cell proliferation, and cell apoptosis (Supplemental Table 2).

### Ch-conditioned media attenuates TGFβ/BMP signaling in both CFLS and JFLS

In an effort to clarify the interaction between FLS and chondrocytes in diseased joints, we exposed FLS to conditioned media containing metabolites, growth factors, and extracellular matrix proteins secreted into the medium by chondrocytes. Following microarray analysis, we examined a curated list of genes related to TGFβ/BMP signaling and calculated their change in expression over 24 h. Examining the log ratios of the fold change over 24 h allowed us to determine changes in gene expression over time that we later correlated to downstream signaling. CFLS-Ch downregulate the gene expression of BMP receptor genes (BMPR1b *p* = 0.003 and BMPR2 *p* = 0.002), BMP ligands (GDF5 *p* = 0.030), and downstream signaling genes (SMAD5 *p* = 0.023 and SMAD7 *p* = 0.035) compared with CFLS (Fig. 2b). JFLS-Ch have significantly decreased gene expression of TGFBI (*p* = 0.016), a gene that induces TGFβ signaling and plays important roles in cell-to-cell, cell-to-collagen, and cell-to-matrix interactions, compared with JFLS [23] (Fig. 2c) (Supplemental Table 3). These findings suggest that exposing FLS to Ch-conditioned media mitigates TGFβ/BMP gene signaling in these cells.

### Ch-conditioned media prevents FLS from differentiating into mature chondrocyte-like cells

Changes in the log ratio of the fold change of linear gene expression over 24 h revealed that CFLS-Ch downregulate genes expressed by hypertrophic chondrocytes and endochondral osteoblasts (COLX *p* = 0.037, VEGFA *p* = 0.034, COL1A1 *p* = 0.031, and COL1A2 *p* = 0.036) compared with CFLS (Fig. 2d). CFLS-Ch overexpress genes that function as proliferation and differentiation factors during EBF (MMP2 *p* = 0.048, FGFR2 *p* = 0.050, and IGF1 *p* = 0.027) compared with CFLS (Fig. 2d) [24–26], suggesting that Ch-conditioned media does not induce CFLS to form mature cartilage. Similarly, JFLS cultured in Ch-conditioned media have decreased gene expression of MMP9 (*p* = 0.038), COL1A1 (*p* = 0.042), SOX6 (*p* = 0.049), and MMP2 (*p* = 0.050) compared with untreated JFLS (Fig. 2e). These genes allow for induction of chondrocyte hypertrophy and permit formation of the prehypertrophic/hypertrophic zone during EBF [6, 18, 27, 28] (Supplemental Table 4). Interestingly, JFLS-Ch significantly upregulate expression of matrillin 3 (MATN3 *p* = 0.023), a gene that inhibits chondrocyte hypertrophy during EBF (Fig. 2e) [29]. Based on our gene expression analysis, it is evident that Ch-conditioned media encourages JFLS to dedifferentiate away from prehypertrophic/hypertrophic chondrocyte-like phenotype.

### Ch influence JFLS to dedifferentiate from mature chondrocyte-like cells by reducing the factors in the joint microenvironment needed to drive EBF

**Fig. 3:**
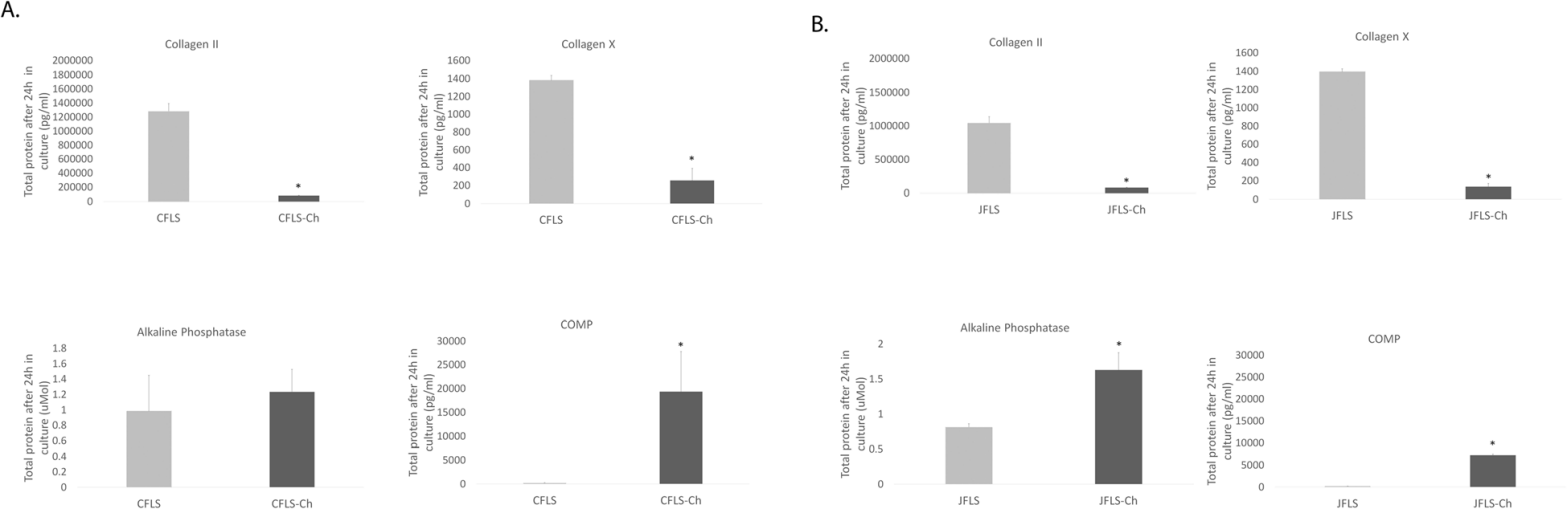
Using ELISA, protein expression was determined for Ch markers. Protein expression of COL2, a marker of proliferating Ch, COLX, a marker of Ch hypertrophy, ALP, a marker of endochondral ossification, and COMP, a marker of late Ch hypertrophy as determined by ELISA on cell media supernatants. The total protein was calculated at 24 h. CFLS-Ch had significant decreases in total protein expression of COL2 (*p* = 0.000) and COLX (*p* = 0.000) when compared with CFLS (**a**). COMP (*p* = 0.009) significantly increases in CFLS-Ch compared to untreated CFLS (**a**). JFLS-Ch significantly downregulate expression of COL2 (*p* = 0.000) and COLX (*p* = 0.000), while ALP (*p* = 0.000) and COMP (*p* = 0.000) expression increases compared with JFLS (**b**)

To determine the changes in JFLS chondrocyte-like phenotype, we used ELISA to measure changes in total protein concentrations after 24 h of the same downstream hypertrophic chondrocyte protein markers (COL2, COLX, COMP, and ALP) when FLS are cultured in Ch-conditioned media. In CFLS-Ch, COL2 (*p* = 0.000) and COLX (*p* = 0.000) significantly decreased compared with untreated CFLS, suggesting that Ch-conditioned media does not promote differentiation of normal FLS along chondrocyte lineage (Fig. 3a). COMP (*p* = 0.009) significantly increased in CFLS cultured in Ch-conditioned media compared to untreated CFLS, suggesting Ch may introduce this marker of cartilage turnover to normal FLS. JFLS cultured in Ch-conditioned media have decreased protein expression of COL2 (*p* = 0.000) and COLX (*p* = 0.000) compared with untreated JFLS (Fig. 3 b). Notably, JFLS-Ch have significantly increased protein expression of ALP (*p* = 0.000) and COMP (*p* = 0.000) compared with JFLS (Fig. 3b). While increases in ALP may suggest corresponding increase in disease activity, reduction of COL2 and COLX protein expression indicates that conditioned media from Ch can influence JFLS to dedifferentiate from the mature chondrocyte-like phenotype.

### JFLS cultured in Ch-conditioned media differentially express BMP antagonists

**Fig. 4:**
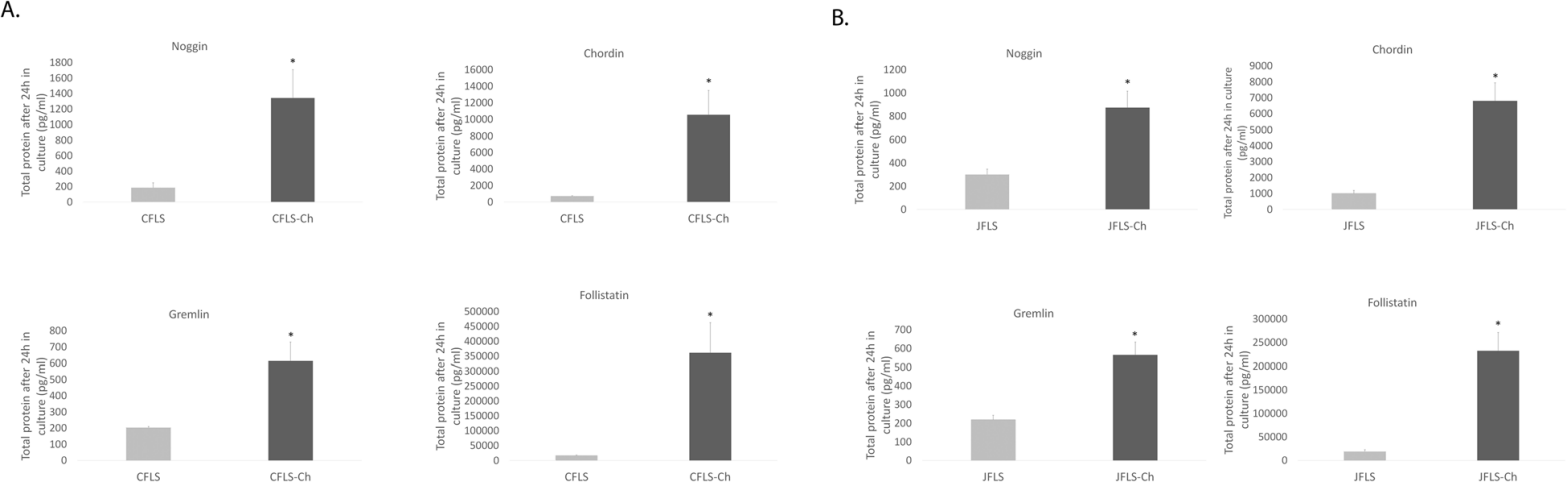
Using ELISA, protein expression was determined for BMP antagonists. CFLS-Ch had significant increases in total protein expressio of BMP antagonists at 24 h (NOG (*p* = 0.002), CHRD (*p* = 0.002), gremlin (GREM) (*p* = 0.001), and follistatin (FST) (*p* = 0.002)) when compared with CFLS (**a**). Conditioned media from Ch influence JFLS to increase total protein amounts of BMP antagonists NOG (*p* = 0.001), CHRD (*p* = 0.000), GREM (*p* = 0.000), and FST (*p* = 0.000) (**b**)

All BMP antagonists had significant increases in absolute protein concentration at 24 h (NOG *p* = 0.002, CHRD p = 0.002, GREM *p* = 0.001, and FST *p* = 0.002) in CFLS-Ch when compared with untreated CFLS (Fig. 4a). JFLS-Ch also significantly increase secretion of BMP antagonist proteins (NOG *p* = 0.001, CHRD *p* = 0.000, GREM *p* = 0.000, and FST *p* = 0.000) when compared with untreated JFLS (Fig. 4b). Taken together, significant decreases in the total protein expression of cell markers of hypertrophic chondrocytes and significant increases in BMP antagonists, thus inhibiting BMP signaling, suggest Ch secrete factors that prevent diseased FLS from differentiating into mature chondrocyte-like cells.

### Exogenous BMP4 has discordant effects on TGFβ protein phosphorylation on FLS cultured in Ch-conditioned media

Previously, we showed that JFLS overexpress BMP4 protein [9]. Additionally, we presented data that suggest JFLS favor BMP4 through increased NOG and CHRD expression and that Ch-conditioned media can upregulate secretion of these antagonists, prompting us to examine the effect of exogenous BMP4 on FLS cultured in Ch-conditioned media. Using protein antibody arrays spotted for phosphorylated proteins specific to TGFβ/BMP signaling (ATF2, c-Fos, c-Jun, SMAD1, SMAD2, SMAD4, SMAD5, and TAK1), we measured the change in intensity in protein phosphorylation at 24 h.

**Fig. 5:**
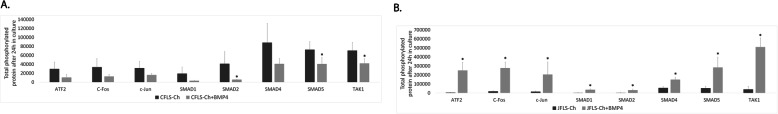
Using antibody arrays spotted for phosphorylated proteins specific to TGFβ/BMP signaling, we measured the total intensity of phosphorylation at 24 h on cell culture supernatants from FLS cultured in conditioned media from Ch (FLS-Ch) and FLS-Ch exposed to exogenous BMP4. CFLS-Ch treated with exogenous BMP4 downregulate the phosphorylation of SMAD2 (*p* = 0.043), SMAD5 (*p* = 0.037), and TAK1 (*p* = 0.040) compared with CFLS-Ch (**a**). JFLS-Ch treated with exogenous BMP4 have increased phosphorylation of ATF2 *p* = 0.000, c-Fos *p* = 0.001, c-Jun *p* = 0.004, SMAD1 *p* = 0.009, SMAD2 *p* = 0.000, SMAD4 *p* = 0.000, SMAD5 *p* = 0.001, and TAK1 *p* = 0.000 compared with JFLS-Ch (**b**)

CFLS cultured in Ch-conditioned media and then treated with exogenous BMP4 have significantly less phosphorylation of proteins SMAD2 (*p* = 0.043), SMAD5 (*p* = 0.037), and TAK1 (*p* = 0.040) when compared with CFLS cultured in Ch-conditioned media alone, suggesting exogenous BMP4 can attenuate TGFβ/BMP protein signaling in CFLS-Ch (Fig. 5a). Conversely, JFLS cultured in Ch-conditioned media and then exposed to exogenous BMP4 have significantly increased protein phosphorylation of all measure proteins (ATF2 *p* = 0.000, c-Fos *p* = 0.001, c-Jun *p* = 0.004, SMAD1 *p* = 0.009, SMAD2 *p* = 0.000, SMAD4 *p* = 0.000, SMAD5 *p* = 0.001, and TAK1 *p* = 0.000) compared with JFLS cultured in Ch-conditioned media alone (Fig. 5b). These findings suggest that the TGFβ/BMP signaling, which is attenuated by BMP antagonists in JFLS cultured in Ch-conditioned media alone, is restored in JFLS-Ch exposed to exogenous BMP4.

### Exogenous BMP4 on JFLS cultured in Ch-conditioned media reestablishes the prehypertrophic/hypertrophic chondrocyte-like phenotype seen in untreated JFLS

**Fig. 6:**
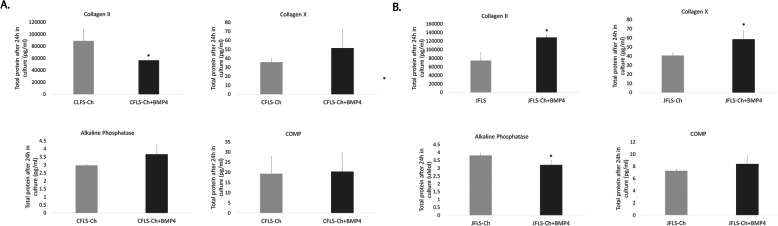
Using ELISA, total protein expression was determined for Ch markers on FLS cultured in Ch-conditioned media and then exposed to exogenous BMP4. Protein expression of COL2, a marker of proliferating Ch, COLX, a marker of Ch hypertrophy, ALP, a marker of endochondral ossification, and COMP, a marker of late Ch hypertrophy as determined by ELISA on cell media supernatants. CFLS-Ch treated with exogenous BMP4 exhibited significant decreases in COL2 (*p* = 0.022) compared with CFLS-Ch (**a**). JFLS-Ch treated with exogenous BMP4 had signifcant increases in COL2 (*p* = 0.004) and COLX (*p* = 0.021) compared with JFLS-Ch, while ALP and COMP remained unchanged (**b**)

Given that expression of TGFβ/BMP signaling genes were downregulated in CFLS-Ch and JFLS-Ch, we measured the influence of exogenous BMP4 on FLS cultured in Ch-conditioned media and its effect on downstream prehypertrophic/hypertrophic chondrocyte protein marker expression using ELISA to calculate total protein at 24 h in culture. COL2 (*p* = 0.022) protein expression significantly decreased in CFLS that were cultured in Ch-conditioned media and treated with exogenous BMP4 compared with CFLS cultured in Ch-conditioned media alone, while COLX, ALP, and COMP remain unchanged in these cells (Fig. 6a). Conversely, exogenous BMP4 in JFLS cultured in Ch-conditioned media caused increased protein expression of both prehypertrophic/hypertrophic markers COL2 (*p* = 0.004) and COLX (*p* = 0.021) compared with JFLS cultured in Ch-conditioned media only, suggesting that even though culturing JFLS in Ch-conditioned media alone eradicated the chondrocyte-like phenotype of diseased FLS, exogenous BMP4 can cause these cells to differentiate as prehypertrophic/hypertrophic chondrocytes (Fig. 6b).

### Inhibition of TGFBR1 does not attenuate TGFβ signaling via SMAD2 phosphorylation in JFLS-Ch

Gene expression analysis revealed that in untreated JFLS, SMAD2 expression was significantly upregulated, suggesting TGFβ activation when compared with untreated CFLS. When JFLS were cultured in Ch-conditioned media, SMAD2 was not significantly different in JFLS-Ch compared with JFLS; however, gene expression did increase over 24 h (Supplemental Table 3). These data prompted us to investigate how the inhibition of TGFBR1 in FLS cultured in Ch-conditioned media would affect TGFβ signaling. Using antibody arrays spotted for phosphorylated proteins specific to TGFβ/BMP signaling (ATF2, c-Fos, c-Jun, SMAD1, SMAD2, SMAD4, SMAD5, and TAK1), we measured the change in intensity in protein phosphorylation after 24 h in culture.

**Fig. 7:**
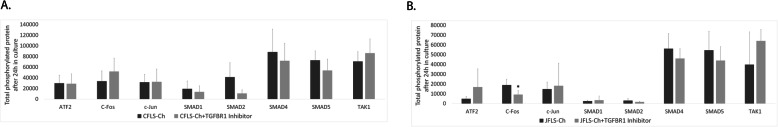
Using antibody arrays spotted for phosphorylated proteins specific to TGFβ/BMP signaling, we measured the total intensity of phosphorylation at 24 h on cell culture supernatants from FLS cultured in conditioned media from Ch and FLS-Ch treated with a TGFBR1 inhibitor. The phosphorylation of ATF2, c-fos, c-Jun, SMAD1, SMAD2, SMAD4, SMAD5, and TAK1 remained unchanged in CFLS-Ch treated with the TGFBR1 inhibitor when compared with CFLS-Ch (**a**). JFLS-Ch treated with the TGFBR1 inhibitor had decreased phosphorylation of c-fos (*p* = 0.041) compared with JFLS-Ch (**b**)

There were no significant differences in the phosphorylation of these proteins when CFLS-Ch were exposed to the TGFBR1 inhibitor compared with CFLS cultured in Ch-conditioned media only (Fig. 7a). JFLS-Ch treated with TGFBR1 inhibitor had significantly less phosphorylation of c-Fos (*p* = 0.042), a TGFβ mediator, compared with JFLS cultured in Ch-conditioned media only (Fig. 7b). While not significant, SMAD2 and SMAD4 have decreased protein phosphorylation in JFLS-Ch treated with TGFBR1 inhibitor compared to JFLS in Ch-conditioned media alone, suggesting that TGFβ signaling via SMAD proteins is downregulated in these cells (Fig. 7b).

### Inhibition of TGFBR1 in JFLS cultured in Ch-conditioned media restores the prehypertrophic/hypertrophic chondrocyte-like phenotype seen in untreated JFLS

**Fig. 8:**
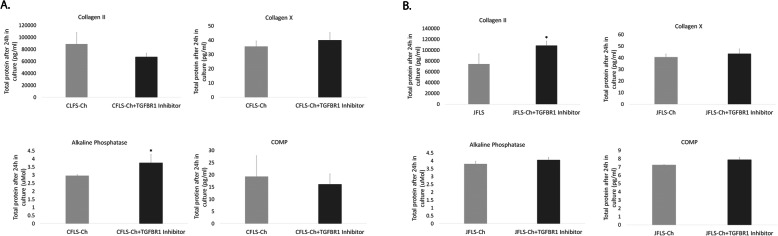
Using ELISA, total protein expression was determined for Ch markers on FLS cultured in Ch-conditioned media and then treated with TGFBR1 inhibitor. Protein expression of COL2, a marker of proliferating Ch, COLX, a marker of Ch hypertrophy, ALP, a marker of endochondral ossification, and COMP, a marker of late Ch hypertrophy as determined by ELISA on cell media supernatants. CFLS-Ch treated with the TGFBR1 inhibitor had increased expression of ALP (*p* = 0.029) compared with CFLS-Ch, while COL2, COLX, and COMP remained unchanged (**a**). JFLS-Ch treated with the TGFBR1 inhibitor had signifcant increases in COL2 (*p* = 0.022) compared with JFLS-Ch, while COLX, ALP, and COMP had no significant changes (**b**)

Quantitative ELISA revealed that ALP (*p* = 0.029) protein expression significantly increases in CFLS that were cultured in Ch-conditioned media and treated with the TGFBR1 inhibitor compared with CFLS cultured in Ch-conditioned media alone, while COLII, COLX, and COMP remained unchanged after 24 h in culture (Fig. 8a). Inhibition of TGFBR1 in JFLS cultured in Ch-conditioned media caused increased protein expression of COL2 (*p* = 0.022) compared with JFLS cultured in Ch-conditioned media alone, suggesting that even though culturing JFLS in Ch-conditioned media alone eradicated the chondrocyte-like phenotype of diseased FLS, inhibition of TGFβR1 can restore expression of a prehypertrophic chondrocyte marker. This response is not as effective as exogenous BMP4 at restoring the chondrocyte-like phenotype of JFLS cultured in Ch-conditioned media.

## Discussion

Consistent with our previously reported observations [9], we show that cultured JFLS have a phenotype that mirrors mature chondrocytes. Specifically, after just 24 h in culture, JFLS have significant increases in gene expression of MMP9, PCNA, and MMP12, reflective of proliferating, prehypertrophic chondrocytes [8]. At confluence, JFLS secrete proteins characteristic of mature and hypertrophic chondrocytes (COL2, COLX, and COMP). While we can characterize JFLS as mimicking chondrocytes in cell culture, we are far from having a thorough understanding of how this cell type contributes to the bony overgrowth observed in affected joints of patients with JIA.

The process of EBF is complex, and many signaling pathways contribute to this process of long bone creation during development. The TGFβ superfamily plays a critical role in EBF, and there is a sophisticated balance between TGFβ and BMP-specific signaling to carry chondrocytes through from proliferation, to hypertrophy, and eventually apoptosis [30]. The data in this study indicate that JFLS have decreased expression of BMP-related genes (BMPR1a and BMP2), prompting us to investigate how JFLS regulate BMP through its antagonists. JFLS secrete significant amounts of noggin and chordin protein, two antagonists with high affinity for BMP4 [31, 32]. Taken together, JFLS in culture are able to create a microenvironment that is favorable for EBF and may play a direct role in the joint growth disturbances seen in affected joints of patients with JIA.

We show, for the first time, an interaction between JFLS and normal chondrocytes using a conditioned-media cell culture model. The data in this study indicate that Ch-conditioned media has an antagonistic effect on the chondrocyte-like phenotype exhibited by JFLS. Microarray analysis revealed a divergent effect on JFLS cultured in Ch-conditioned media when compared with JFLS cultured in their respective media. Specifically, Ch-conditioned media attenuated TGFβ signaling through a significant decrease in TGFβ-inducing gene. Furthermore, genes expressed by hypertrophic chondrocytes are also downregulated in JFLS-Ch, while MATN3, an inhibitor of chondrocyte hypertrophy during EBF [29], is overexpressed in JFLS-Ch. When examining the protein secretion profile of JFLS cultured in Ch-conditioned media, chondrocytes influence JFLS to decrease the amount of COL2, COLX, and COMP, prominent markers of mature and hypertrophic chondrocytes. This fluidity of the abnormal chondrocyte-like phenotype seen in JFLS thus far could be attributed to the pluripotent properties of FLS and cell plasticity that allows them to acclimate to influences in their culture environment, namely media containing growth factors and metabolites from normal chondrocytes.

Another possibility is that media from normal chondrocytes can influence JFLS to revert to a proliferative state, as indicated by a significant upregulation of BMP antagonists noggin, chordin, gremlin, and follistatin. A study by Keller et al. indicates that TGFβ is favored by proliferating and prehypertrophic chondrocytes, and that the crosstalk between TGFβ and BMP results in a decrease in TGFβ signaling instead of an increase in BMP signaling as chondrocytes progress through EBF [30].

Given that JFLS differentiate down a chondrocyte lineage and that conditioned media from chondrocytes can abrogate this phenotype, we examined the mechanism by which TGFβ and BMP can influence the altered population of JFLS-Ch. Exogenous BMP4 can cause JFLS-Ch to increase phosphorylation of BMP proteins and promote secretion of COL2 and COLX. BMP4 promotes hypertrophy in chondrocytes [31] and appears to be having the same effect on JFLS cultured in Ch-conditioned media further supporting the notion that BMP is favored by cells undergoing hypertrophy during EBF [33].

Inhibiting TGFβR1 had a similar effect as exogenous BMP4 on JFLS cultured in Ch-conditioned media. These cells significantly increased secretion of COL2. On the basis of our aforementioned data, we postulate that JFLS-Ch emulate proliferating/prehypertrophic chondrocytes and that exposing these cells to a TGFβR1 inhibitor promotes crosstalk between signaling pathways, allowing for unencumbered BMP signaling to differentiate JFLS cultured in Ch-conditioned media toward hypertrophy.

Exposing JFLS cultured in Ch-conditioned media to exogenous BMP4 elicited a robust restorative response of chondrocyte-like phenotype seen in untreated JFLS, suggesting this growth factor may translate to clinical implications. Based on our findings, we conclude that BMP4 influences the cell culture microenvironment of JFLS. Further studies examining levels of BMP4 in affected joint synovial fluid and the inhibition of BMP4 would need to be conducted. Increased BMP4 seen in untreated JFLS suggests that blocking this growth factor in vivo could prevent the hypertrophy of these cells and possibly prevent joint growth disturbances seen in JIA.

We recognize there are limitations to this study, namely our sample size and isolating FLS and Ch in culture. We must consider the possibility that using normal primary adult Ch could contribute to an age-related response. There are many contributing influences in vivo that contribute to the pathogenesis of JIA, and it is known that inflammatory factors can promote EBF; however, we have established a contributing role of JFLS in joint growth disturbances of affected joints.

## Conclusions

In conclusion, JFLS in vitro have a prehypertrophic/hypertrophic chondrocyte-like phenotype and produce a microenvironment favorable for EBF. Conditioned-media from normal chondrocytes can deconstruct this microenvironment by attenuating TGFβ/BMP gene expression and BMP antagonist protein expression. Chondrocytes may influence JFLS to dedifferentiate toward a proliferative state. Specifically, exogenous BMP4 or the inhibition of TGFβR1 can overcome this influence of chondrocytes on JFLS, and JFLS resume a phenotype similar to hypertrophic chondrocytes. Our data are the first to isolate this interaction between two prominent cell types found in the joints of patients with JIA. Additionally, we propose a novel role for JFLS, suggesting that these cells, without being reliant on chondrocytes, could contribute directly to EBF based on their chondrocyte-like phenotype and the regulation of TGFβ/BMP in these cells. Lastly, this unique behavior of JFLS could explain a possible process and mechanism for which joint growth disturbances occur in joints of patients with JIA.

## Supplementary Information

**Additional file 1: Table 1.** Differentially expressed genes with a 1%FDR. Utilizing an unbiased approach to globally characterize FLS and FLS cultured in conditioned media from Ch, we discovered distinct discordances in gene expression. LIMMA analysis revealed 246 genes differentially expressed in CFLS vs CFLS cultured in conditioned media from chondrocytes (CFLS-Ch) and 31 genes differentially expressed in JFLS vs conditioned media from chondrocytes (JFLS-Ch) after 6 h in culture (1% FDR).

**Additional file 2: Table 2.** Top ‘Ready-Analysis’ genes for both untreated FLS and FLS cultured in conditioned media from chondrocytes. LIMMA was performed on all 21,448 transcript clusters included on Clariom S Array. Table includes gene symbol of the top ‘ready-analysis’ genes as determined by Ingenuity Pathway Analysis for untreated FLS and FLS-Ch after LIMMA analysis was performed to determine differentially expressed genes with a 1% FDR. Additionally, genes are listed that regulate these genes and genes that are regulated by the top ‘ready-analysis’ genes.

**Additional file 3: Table 3.** Curated list of TGFβ/BMP signaling genes. Table contains the list of 27 genes specific to TGFβ/BMP signaling. This list was generated using Ingenuity Pathway Analysis (IPA). Differentially expressed genes with an FDR of 1% were input into IPA and TGFβ/BMP pathway and top ‘ready-analysis’ genes were related to this signaling pathway. Based on this finding, we analyzed these genes using Excel and provided the averages, standard deviations, and *p*-values for all genes analyzed in this table.

**Additional file 4: Table 4.** Curated list of genes related to chondrocyte proliferation, maturation, and hypertrophy. This list was generated using Ingenuity Pathway Analysis (IPA). Differentially expressed genes with an FDR of 1% were input into IPA and top networks pertaining to cell differentiation were examined. A list was curated for genes specific to chondrocytes. We analyzed these genes using Excel and provided the averages, standard deviations, and *p*-values for all genes analyzed in this table.

## Data Availability

The microarray datasets used and/or analyzed during the current study are available on GEO using accession number GSE165626. The ELISA and phosphorylation protein assay datasets are available on FigShare using the following link: https://figshare.com/s/36109a85c13ac0fbf259 License: CC BY 4.0.

## Abbreviations

JIA: Juvenile Idiopathic Arthritis
FLS: Fibroblast-like Synoviocytes
JFLS: JIA FLS
BMP4: Bone Morphogenetic Protein 4
EBF: Endochondral bone formation
Ch: Chondrocytes
CFLS: Control FLS
COL2: Collagen II
COLX: Collagen X
ALP: Alkaline Phosphatase
CFLS-Ch: CFLS with Ch-conditioned media
JFLS-Ch: JFLS with Ch-conditioned media
