# Supplementary material for: The culture microenvironment of juvenile idiopathic arthritis synovial fibroblasts is favorable for endochondral bone formation through BMP4 and repressed by chondrocytes
Source: Pediatr Rheumatol Online J. 2021 May 12;19:72. doi: 10.1186/s12969-021-00556-8 (PMC8117630; doi:10.1186/s12969-021-00556-8)
Supplement: Supplementary file 1 — Additional file 1: Table 1. Differentially expressed genes with a 1%FDR. Utilizing an unbiased approach to globally characterize FLS and FLS cultured in conditioned media from Ch, we discovered distinct discordances in gene expression. LIMMA analysis revealed 246 genes differentially expressed in CFLS vs CFLS cultured in conditioned media from chondrocytes (CFLS-Ch) and 31 genes differentially expressed in JFLS vs conditioned media from chondrocytes (JFLS-Ch) after 6 h in culture (1% FDR). [file 12969_2021_556_MOESM1_ESM.pdf]

Transcript Cluster ID	CFLS v CFLS-Ch
TC2200009231.hg.1	-2.200382326
TC1200006649.hg.1	-1.898279294
TC1200010016.hg.1	1.472368412
TC0700011119.hg.1	-1.881196204
TC1000008092.hg.1	-1.403097564
TC0100015754.hg.1	3.694566569
TC1500009204.hg.1	1.239332881
TC0300010772.hg.1	3.053852823
TC0100012473.hg.1	-1.818672214
TC0500009629.hg.1	-1.600840068
TC1500010783.hg.1	-2.002062231
TC1700011919.hg.1	1.791767193
TC1200010615.hg.1	-2.499819738
TC1600011373.hg.1	-1.91210333
TC0X00007709.hg.1	-1.864626565
TC1100010505.hg.1	2.785804758
TC1200012265.hg.1	-1.466157468
TC1700011984.hg.1	4.511070936
TC0400007556.hg.1	-1.709716885
TC1200012716.hg.1	-1.416215437
TC0100011276.hg.1	-1.469773685
TC1400010615.hg.1	-1.407797504
TC0100008152.hg.1	-1.322162373
TC0200010837.hg.1	-1.955483886
TC0900012167.hg.1	2.240489131
TC1500008988.hg.1	-1.999681658
TC0200016452.hg.1	3.442244092
TC0700006913.hg.1	2.56445914
TC0100013873.hg.1	2.787676111
TC1600011372.hg.1	-1.586195994
TC1000012589.hg.1	-1.552391595
TC2200009257.hg.1	2.107148117
TC1100011797.hg.1	2.300820271
TC1000011718.hg.1	-1.7085525
TC0400008663.hg.1	2.749392807
TC1500008995.hg.1	-1.83107136
TC1600011398.hg.1	1.015571906
TC0100015810.hg.1	-1.442598121
TC0900009932.hg.1	-1.467585429
TC1200009217.hg.1	-0.945389969
TC1900009769.hg.1	-1.567948174
TC1300008272.hg.1	1.731959222
TC0X00011163.hg.1	-1.553439998
TC0700012761.hg.1	3.87979084
TC0800011561.hg.1	-0.97193856
TC1900008498.hg.1	0.974710638

Transcript Cluster ID	JFLS v JFLS-Ch
TC1200008659.hg.1	1.62700342
TC1300010011.hg.1	1.857657576
TC1000010911.hg.1	1.686409221
TC1200010752.hg.1	1.61610607
TC1900008513.hg.1	1.072420644
TC0500008619.hg.1	1.635871215
TC1000011287.hg.1	1.150359876
TC1100011485.hg.1	2.19613468
TC1500009149.hg.1	-1.4559126
TC0200016623.hg.1	1.126871312
TSUnmapped00000174.hg.1	1.930870915
TC1600007424.hg.1	1.555994579
TC1100006456.hg.1	1.372019861
TC1900010716.hg.1	-1.335991304
TC0500010478.hg.1	1.178766614
TC1700009037.hg.1	1.211218169
TC1400008908.hg.1	-1.771956909
TC1200011755.hg.1	-1.858169301
TC1000007905.hg.1	1.028050682
TC1900011997.hg.1	1.355431834
TC0400007784.hg.1	1.268541233
TC0700008963.hg.1	1.418600321
TC0700008454.hg.1	1.364918322
TC1600007251.hg.1	1.335343902
TC0800010158.hg.1	-1.265213176
TC0300011517.hg.1	1.421718517
TC1100009924.hg.1	1.498780062
TC2200008008.hg.1	1.278408994
TC1200010615.hg.1	-2.015574501
TSUnmapped00000016.hg.1	1.339356261
TC0900010922.hg.1	-1.263562657

TC1200007866.hg.1	-1.369356709
TC2200006601.hg.1	-1.327423344
TC1600008675.hg.1	2.138814537
TC1300008716.hg.1	1.488811962
TC0200008291.hg.1	-2.003022575
TC0100009211.hg.1	-1.426627514
TC2000009882.hg.1	0.903011192
TC0200016787.hg.1	-1.247884552
TC0100010791.hg.1	1.145796385
TC1400010629.hg.1	3.420333918
TC0900011379.hg.1	1.842972941
TC0700008522.hg.1	-1.235678279
TC0300006968.hg.1	-2.608355362
TC0500007465.hg.1	1.774034164
TC2000006781.hg.1	-0.965920987
TC0700013620.hg.1	-1.790684647
TC0400008882.hg.1	1.589766036
TC0400012057.hg.1	-1.238778894
TC0400008362.hg.1	-1.525607055
TC0300011306.hg.1	-1.277678481
TC1000012329.hg.1	-2.008556757
TC0300012186.hg.1	-1.502013397
TC0100017945.hg.1	-1.254828847
TSUnmapped00000351.hg.1	1.013886696
TC1600011566.hg.1	-1.128721977
TC1500008225.hg.1	-1.588763498
TC0700007392.hg.1	1.519352381
TC0500009559.hg.1	-1.635855038
TC2000008955.hg.1	-0.921736881
TC2200009275.hg.1	-1.299371425
TC0100013925.hg.1	-1.222424609
TC0700012512.hg.1	1.851841914
TC0200016654.hg.1	-1.35073401
TC1100008175.hg.1	-1.56141062
TC2000008007.hg.1	-0.975631269
TC1700011233.hg.1	-1.803963638
TC2200008346.hg.1	3.155667082
TC2200008203.hg.1	-1.43851327
TC0200016732.hg.1	-1.269926191
TC1100009981.hg.1	1.29386428
TC2000007227.hg.1	-1.313445596
TC0100018438.hg.1	2.151253545
TC1900007688.hg.1	-1.511850482
TC1200009735.hg.1	-1.208054734
TC0500008229.hg.1	1.991288494
TC1100012315.hg.1	-1.171704747
TC1000010452.hg.1	-1.39255637

TC1700006546.hg.1	-1.530565886
TC1400010339.hg.1	-1.119096316
TC1100013147.hg.1	-1.026298629
TC0100018237.hg.1	-1.138242568
TC0100018435.hg.1	-1.52786304
TC0600007613.hg.1	3.098862958
TC0400008271.hg.1	2.581056119
TC1700012309.hg.1	-1.234968048
TC0X00007668.hg.1	-1.103101499
TC0200009281.hg.1	-1.235342221
TC1000007092.hg.1	2.701463239
TC0500007668.hg.1	-1.576577063
TC0900012212.hg.1	-3.353645061
TC0100008321.hg.1	-1.92047825
TC0100016431.hg.1	2.104056245
TC1700010190.hg.1	-1.466266886
TC1100007940.hg.1	1.56157901
TC1900008300.hg.1	1.887370893
TC0300013813.hg.1	1.488741579
TC0100006840.hg.1	-0.94598086
TC1500008984.hg.1	-1.905570306
TC1500009283.hg.1	1.621320695
TC1900011651.hg.1	1.105297822
TC0200006440.hg.1	-1.630511409
TC0100013709.hg.1	1.486977125
TC1700006702.hg.1	-1.245472484
TC1900008057.hg.1	2.221527551
TC0700008190.hg.1	2.338468793
TC1900006795.hg.1	-1.252375462
TC0400011144.hg.1	1.949997206
TC1900010523.hg.1	-1.018152414
TC1100012526.hg.1	2.950482226
TC2000007792.hg.1	-1.263811691
TC0200015216.hg.1	-1.389516154
TC0200013079.hg.1	-1.502842012
TC0700006484.hg.1	-1.053455645
TC0900006481.hg.1	-2.370436181
TC0500013245.hg.1	1.806991585
TC1200009172.hg.1	-1.849158619
TC1400010636.hg.1	-1.987218293
TC0200006536.hg.1	-1.000442081
TC0100015352.hg.1	-1.703303027
TC0800007316.hg.1	-2.442078722
TC1600007513.hg.1	-1.324292166
TC0500010999.hg.1	-1.076005296
TC0400012891.hg.1	1.229658142
TC0600007616.hg.1	2.139029448

TC1100013185.hg.1	-1.489919206
TC1900011707.hg.1	-0.964085808
TC1800008162.hg.1	-1.698588026
TC1500010658.hg.1	-1.499951959
TC1100007210.hg.1	-0.893371227
TC1900008103.hg.1	1.434537772
TC0600007012.hg.1	2.82094932
TC1600008906.hg.1	-1.221386111
TC1700012477.hg.1	1.51766862
TC1100007408.hg.1	1.366580952
TC1200007710.hg.1	-1.124822766
TC0200013112.hg.1	-1.206111544
TC1700012468.hg.1	-1.975425128
TC0100011552.hg.1	1.603298664
TC0500011702.hg.1	-1.616717398
TC0300013847.hg.1	-1.638168537
TC0200009134.hg.1	-1.442859054
TC0600014237.hg.1	-1.113947619
TC2200009351.hg.1	-1.080217793
TC1000007199.hg.1	1.664433444
TC1700006464.hg.1	-1.874166444
TC1200012206.hg.1	-1.681627811
TC0800010154.hg.1	-1.135812105
TC0300008989.hg.1	-1.783039091
TC0100017142.hg.1	3.463267411
TC1700011128.hg.1	0.980293215
TC0700009134.hg.1	-2.533855065
TC0500012282.hg.1	-0.781090131
TC1200009964.hg.1	1.671632303
TC0500009614.hg.1	-1.527329075
TC1500009804.hg.1	1.123372088
TC0100011767.hg.1	-1.821997004
TC1200012721.hg.1	-1.378500748
TC0700013596.hg.1	-1.074228168
TC0500008653.hg.1	-1.670974686
TC1100009237.hg.1	1.138098215
TC1700006644.hg.1	-1.613481474
TC0400011053.hg.1	7.716931464
TC0500009787.hg.1	1.123526632
TC2200007361.hg.1	-1.612025462
TC2200006641.hg.1	-1.212454294
TC0100013756.hg.1	-1.628167742
TC0700008494.hg.1	-1.125440323
TC1900011857.hg.1	-1.762341421
TC1900010856.hg.1	-1.502315322
TC1800008231.hg.1	-0.958642291
TC0100015781.hg.1	0.993709483

TC0300010213.hg.1	-1.179090469
TC2200009278.hg.1	-1.376064671
TC1000009172.hg.1	-1.190252634
TC1600011513.hg.1	-1.548381107
TC1700010084.hg.1	-1.003912693
TC0300012718.hg.1	-1.745479914
TC1700009236.hg.1	-2.439219793
TC0700009872.hg.1	-1.546452086
TC1000008575.hg.1	-0.811166417
TC1900006890.hg.1	-1.358465176
TC0200010567.hg.1	-1.203383609
TC2000010022.hg.1	2.391730905
TC1900006520.hg.1	-1.999549839
TC0500008698.hg.1	1.263842909
TC0200007205.hg.1	2.290203784
TC1100012535.hg.1	2.083317938
TC1700007017.hg.1	-1.325037617
TC0300011801.hg.1	-1.582099353
TC1700007890.hg.1	1.489737165
TC0300013804.hg.1	-1.480297079
TC1600009252.hg.1	2.370361393
TC2200008829.hg.1	-1.112600708
TC1300007780.hg.1	-1.105661491
TC0200015211.hg.1	1.581928753
TC1200009131.hg.1	-1.00873999
TC1100009904.hg.1	-0.972570992
TC1200012163.hg.1	-1.322394976
TC0900011428.hg.1	-1.208239269
TC2200008734.hg.1	1.208560339
TC1100007301.hg.1	-2.820304495
TC1700012058.hg.1	-1.400495412
TC0900009106.hg.1	-0.995276234
TC1400007207.hg.1	1.303663915
TC2200007035.hg.1	-1.016950808
TC0600014045.hg.1	-0.971854204
TC1000008396.hg.1	8.935948449
TC0500012163.hg.1	-1.368424069
TC1900008013.hg.1	-1.083702833
TC0900011496.hg.1	-0.863278743
TC0900006442.hg.1	1.871286202
TC1100012514.hg.1	-1.125424323
TC0300013833.hg.1	-1.214145618
TC0600011357.hg.1	2.115894835
TC0100015355.hg.1	-1.421176115
TC0800010551.hg.1	1.580471095
TC0600008120.hg.1	1.71415901
TC0700012427.hg.1	0.938257915

TC0X00007690.hg.1	-1.000085035
TC0700011847.hg.1	-1.467190241
TSUnmapped00000195.hg.1	-1.145638089
TC0700011603.hg.1	1.657570258
TC2200007963.hg.1	1.302221518
TC0500012354.hg.1	-1.018438265
TC1000009090.hg.1	1.382738375
TC1400007548.hg.1	2.201243633
TSUnmapped00000077.hg.1	-1.458785308
TC1100006644.hg.1	1.327592568
TC0100010672.hg.1	1.118717332
TC0600011560.hg.1	-1.676790997
TC1000012510.hg.1	-1.530688959
