# Supplementary material for: The culture microenvironment of juvenile idiopathic arthritis synovial fibroblasts is favorable for endochondral bone formation through BMP4 and repressed by chondrocytes
Source: Pediatr Rheumatol Online J. 2021 May 12;19:72. doi: 10.1186/s12969-021-00556-8 (PMC8117630; doi:10.1186/s12969-021-00556-8)
Supplement: Supplementary file 2 — Additional file 2: Table 2. Top ‘Ready-Analysis’ genes for both untreated FLS and FLS cultured in conditioned media from chondrocytes. LIMMA was performed on all 21,448 transcript clusters included on Clariom S Array. Table includes gene symbol of the top ‘ready-analysis’ genes as determined by Ingenuity Pathway Analysis for untreated FLS and FLS-Ch after LIMMA analysis was performed to determine differentially expressed genes with a 1% FDR. Additionally, genes are listed that regulate these genes and genes that are regulated by the top ‘ready-analysis’ genes. [file 12969_2021_556_MOESM2_ESM.pdf]

Top 'Ready-Analysis' Genes		Gene	Regulates	Regulated by
	CFLS			
		IFIT2	TNF, IL6, IL12B, IL1B, PRKCI, IRF3	IFNB1, IFNA2, STAT1, TICAM1, STAT2
		CXCL10	CXCR3, MMP9, ERK1/2, AKT, IL6, PI3K, CXCL10, IL1A, IL1B, TNF, TGFB1, IL2, CXCL9	TNF, IL1B, STAT1, TLR4, TLR3, IL10, IFNB1, TICAM1
		NPTX1	KCNQ2, SYP, DLG4, VEGFA, KDR, BAX	HTT, JAK1/2, CARD9, SNCA, CTNNB1
		ZC3HAV1	IL6, IFNA1, CXCL10, IFNA4, TNF, IRF3	EIF2AK2, NKX2-3, PAF1, MAPK1
		CTSK	COL1A1, COL2, ACP5, APOB, CXCL11, CXCL10, CXCL9, CXCL8, CXCL5, CXCL3, CXCL2	TNFSF11, CSF1, MITF, NPC1, IL13, IL6, TGFB1, IL4, NFATC1
	CFLS-Ch			
		PLIN2	LEP, PLIN3, UCP1, UCP2, PCK1	PPARD, PPARG, PPARA, SCP2
		SLC16A3	IL12, CASP3, MMP9, IL1B, NOS2, TNF, IL6	HIF1A, MYC, IKBKE, MAPT, EGLN, CSF1, IL15
		VLDLR	DAB1, SLC38A5, TNFAIP3, SLC2A1, APOB, ABCA1	MYLIP, PPARG, PLN, NFAT5
		MIF	TNF, IL1B, JUN, TLR4, ERK1/2, CXCL8, IL6, TIMP1, CCL2, CCND1, MMP1, IL17A	HIF1A, IL1B, AGT, IFNG, MYC, TNF, P38 MAPK, TGFB, IL15
		BNIP3	BCL2L1, GABARAPL2, MAP1LC3B, AIFM1, BCL2, RHEB, EIF4EBP1	FOXO3, HIF1A, TP53, E2F1, EGLN, TRPM2, MAPK, SIRT1
	JFLS			
		SHANK2	SLC9A3, CFTR, GRIN2, NCF1	SOD1, HOXA3, RUNX1, RARA, ESR1, MAPK1, CYP1B1, CYP1A1, CYP1A2, SSTR2, SST
		BCL2L14	MELK, BCL2L14, MAPK1, ERK, ERK1/2, TNF	DBP, TEF, IFNA2, IFNAR1, IFNAR2, MELK
		IL3	BCL2L1, STAT5, CISH, MYC, PIM1, CSF1R, FOS, CD40, JAK2, CD80, BCL2, IL3RA, SLC2A1	IL1, CD28, IL4, CD3, IL12, RUNX1, TNF, BCL2
		ZG16	IL6, IFNG	ARNT2, SIM1
		PROK2	PROKR2, STAT3, HIF1A, CDC25A, BCL2, FOS, VEGFA, IL1B, PROKR1, AKT, NFATC3	STAT3, CSF3, HTT, TNF, CSF2, STAT5, STAT1, ERK1, ERK2, IL6, IL2
	JFLS-CH			
		LMBR1	MAGEA11, PTPRN, TCTN3, EVC2, TCTN2, CREB3, LCN1	NRDE2
		NFYB	FGFR2, DSPP, PCK1, BCL2L1, COL1A1, SNAI1, CDH2, CDH1, TJP1, TWIST1, ACTA2, CTNNB1	ESR1, BMP2, GCG, NCAM1, IL1B
		NDUFAF1	ECSIT, NDUFS3, ACAD9, TMEM126B, TIMMDC1, HSPD1, PPP2R2D, NDUFA13, NDUFA8, LPAR1, FAS	NR2C2, TLE3, SOD1, FOXO1, TP53
		COQ8B	P2RX4, CD27, TGFBR2, RNF123, EGLN3, SLC2A12, APLNR, CD70, VIPR2, PITRM1, COQ5, PMPCA, ATP5PB	NR3C1
		ZNF510	STK11, TGFBR2, SMAD2, EGFR, CCND1, APC, PTPRJ, CDH1	BRAF, BUB1, DLC1, PDGFRL, FBXW7, MLH3, CTNNA1
