# Supplementary material for: The culture microenvironment of juvenile idiopathic arthritis synovial fibroblasts is favorable for endochondral bone formation through BMP4 and repressed by chondrocytes
Source: Pediatr Rheumatol Online J. 2021 May 12;19:72. doi: 10.1186/s12969-021-00556-8 (PMC8117630; doi:10.1186/s12969-021-00556-8)
Supplement: Supplementary file 3 — Additional file 3: Table 3. Curated list of TGFβ/BMP signaling genes. Table contains the list of 27 genes specific to TGFβ/BMP signaling. This list was generated using Ingenuity Pathway Analysis (IPA). Differentially expressed genes with an FDR of 1% were input into IPA and TGFβ/BMP pathway and top ‘ready-analysis’ genes were related to this signaling pathway. Based on this finding, we analyzed these genes using Excel and provided the averages, standard deviations, and p-values for all genes analyzed in this table. [file 12969_2021_556_MOESM3_ESM.pdf]

Gene	Averages	CFLS	CFLS-Ch	JFLS	JFLS-Ch	Standard Deviation	CFLS	CFLS-Ch	JFLS	JFLS-Ch	p-value	CFLS v JFLS	CFLS v CFLS-Ch	JFLS v JFLS-Ch
Smad1		0.203	0.163	0.235	-0.070		0.089	0.487	0.424	0.158		0.452	0.448	0.154
Smad5		0.208	0.094	0.061	-0.001		0.043	0.054	0.142	0.050		0.081	0.023	0.256
Smad9		0.808	0.568	0.690	0.413		0.158	0.159	0.307	0.380		0.294	0.069	0.191
Smad2		0.031	0.014	0.124	0.147		0.015	0.059	0.048	0.039		0.016	0.327	0.286
Smad4		0.089	0.130	0.084	0.099		0.048	0.038	0.093	0.122		0.469	0.154	0.437
Smad6		1.152	1.017	1.103	0.947		0.288	0.180	0.452	0.327		0.441	0.266	0.327
Smad7		0.081	-0.239	-0.039	-0.250		0.165	0.150	0.290	0.090		0.283	0.035	0.148
Smad3		-0.244	-0.533	-0.445	-0.715		0.230	0.277	0.339	0.295		0.222	0.118	0.178
SKI		0.443	0.375	0.345	0.432		0.244	0.183	0.276	0.144		0.336	0.361	0.327
Smurf1		-0.195	-0.195	-0.236	-0.381		0.075	0.058	0.020	0.178		0.206	0.500	0.116
Smurf2		-0.147	0.042	0.035	0.162		0.363	0.163	0.416	0.505		0.300	0.229	0.377
BMP3		-0.038	-0.048	0.025	0.080		0.141	0.084	0.143	0.125		0.308	0.460	0.321
BMPR1a		0.451	0.374	0.246	0.377		0.076	0.117	0.034	0.208		0.007	0.199	0.170
BMP1		0.214	0.510	0.187	0.112		0.055	0.077	0.028	0.108		0.245	0.003	0.156
bmpr1b		0.023	-0.409	0.112	0.056		0.087	0.120	0.333	0.158		0.338	0.003	0.402
bmp2		-0.257	-0.404	-0.957	-0.349		0.240	1.090	0.460	0.720		0.041	0.415	0.143
bmpr2		0.399	0.069	0.179	0.140		0.084	0.056	0.183	0.042		0.065	0.002	0.372
bmp4		0.304	0.781	0.551	0.156		0.417	0.875	0.642	0.169		0.303	0.221	0.180
tgfb1		0.493	0.399	0.285	0.414		0.157	0.299	0.077	0.230		0.054	0.328	0.204
tgfbr1		-0.075	-0.279	-0.173	-0.251		0.330	0.208	0.258	0.285		0.354	0.208	0.371
tgfb2		-0.149	-0.264	-0.476	-0.538		0.287	0.560	0.162	0.448		0.080	0.383	0.416
tgfbr2		-0.313	-0.147	-0.151	-0.102		0.038	0.175	0.209	0.287		0.128	0.091	0.413
tgfb3		-0.274	-0.716	-0.612	-1.025		0.257	0.305	0.477	0.251		0.170	0.064	0.128
tgfbr3		-0.286	-0.032	0.041	0.137		0.221	0.120	0.398	0.438		0.141	0.078	0.396
tgfbi		0.087	0.076	0.143	-0.029		0.072	0.116	0.067	0.060		0.189	0.448	0.016
bmp7		-0.070	-0.036	0.033	-0.078		0.131	0.052	0.078	0.096		0.153	0.349	0.097
gdf5		0.457	0.005	0.201	-0.181		0.207	0.219	0.521	0.560		0.237	0.030	0.219
