# Supplementary material for: The culture microenvironment of juvenile idiopathic arthritis synovial fibroblasts is favorable for endochondral bone formation through BMP4 and repressed by chondrocytes
Source: Pediatr Rheumatol Online J. 2021 May 12;19:72. doi: 10.1186/s12969-021-00556-8 (PMC8117630; doi:10.1186/s12969-021-00556-8)
Supplement: Supplementary file 4 — Additional file 4: Table 4. Curated list of genes related to chondrocyte proliferation, maturation, and hypertrophy. This list was generated using Ingenuity Pathway Analysis (IPA). Differentially expressed genes with an FDR of 1% were input into IPA and top networks pertaining to cell differentiation were examined. A list was curated for genes specific to chondrocytes. We analyzed these genes using Excel and provided the averages, standard deviations, and p-values for all genes analyzed in this table. [file 12969_2021_556_MOESM4_ESM.pdf]

Averages

Gene	CFLS	CFLS-Ch	JFLS	JFLS-Ch
Sox9	-0.251862	-0.252865	-0.431476	-0.930183
Runx2	1.064643	0.453552	0.6723	0.388329
ColX	0.022076	-0.114391	0.122419	-0.019065
MMP9	-0.071244	0.028096	0.228783	-0.145498
MMP13	0.130446	0.264906	0.011038	0.199683
ADAMTS5	-0.299023	-0.075257	-0.40037	0.063216
ColIX	-0.053182	-0.186639	0.055189	0.183628
ColXI	0.708424	0.321099	0.043148	-0.238817
ColVI	-0.006021	-0.019065	0.001003	0.061209
MATN1	-0.014048	-0.073251	-0.045154	-0.042144
Ihh	-0.056192	0.020069	-0.018062	0.055189
VEGFA	-0.153525	-0.592026	-0.586005	-0.429469
SPP1	0.238817	0.052179	-0.207711	-0.043148
ALK1	-0.045154	-0.119409	-0.075257	0.115395
MMP3	0.196673	0.743544	-0.044151	0.115395
Ki67	-0.536837	-0.858939	-0.50272	-0.515765
PCNA	-0.191656	0.129443	0.219752	0.188645
MATN3	0.041141	0.031106	-0.075257	-0.007024
Pth1r	0.111381	0.087299	0.018062	0.030103
b-catenin	0.125429	0.123422	0.116398	-0.090309
Runx3	-0.075257	-0.028096	-0.03512	-0.03813
Foxa2	0.039134	0.065223	-0.031106	-0.162556
Mef2c	-0.328123	-0.294006	-0.006021	-0.558912
RANKL	0.071244	0.547875	0.145498	0.396356
Collagen II	0.13145	0.033113	0.141484	0.132453
Col1a1	0.310061	-0.125429	0.243834	-0.080275
Col1a2	0.040137	-0.133457	0.056192	-0.047161
Sox5	0.36525	0.717455	0.562926	0.443518
Sox6	0.482651	0.321099	0.443518	0.151518
MMP2	0.012041	0.159546	0.081278	-0.022076
MMP12	-0.045154	-0.021072	0.127436	-0.03211
Aggrecan	0.322102	-0.218748	0.011038	-0.279958
Nkx3-2	-0.004014	-0.122419	0.045154	0.10536
GATA4	-0.108371	-0.048165	-0.115395	-0.010034
GATA5	0.0291	0.006021	-0.006021	0.027093
GATA6	-0.081278	-0.030103	0.155532	0.037127
hif1a	0.075257	0.140481	0.049168	0.012041
hdac4	0.076261	0.182625	0.275944	0.103354
sik3	-0.503724	-0.270927	-0.322102	-0.257882
fgfr1	-0.275944	-0.398363	-0.172591	-0.297016
fgfr2	-0.015051	0.19868	-0.072247	-0.060206
fgfr3	0.116398	0.069237	0.083285	0.039134
fgf9	0.054185	-0.13446	0.114391	0.250858
fgf18	0.175601	0.196673	0.121415	0.128439
snail1	0.46459	-0.334143	-0.394349	-0.390336

STD

CFLS	CFLS-Ch	JFLS
0.378991	0.494569	0.70598
0.27834	0.41708	0.479388
0.089012	0.041712	0.296332
0.134972	0.323454	0.164082
0.169898	0.534436	0.125726
0.249952	0.649995	0.33375
0.166476	0.140906	0.441205
0.301246	0.739152	0.012166
0.069433	0.124494	0.022189
0.081464	0.101877	0.086359
0.13917	0.033159	0.101773
0.253205	0.173094	0.151126
0.314808	0.223581	0.173661
0.042466	0.043241	0.174675
0.452991	1.346826	0.161559
0.196047	0.509607	0.634188
0.12691	0.314414	0.10467
0.228155	0.384204	0.031428
0.12654	0.143013	0.070405
0.137598	0.140906	0.168263
0.334794	0.041092	0.015152
0.03948	0.043865	0.045288
0.107742	0.314779	0.665126
0.127195	0.865858	0.219594
0.319891	0.084771	0.02464
0.223432	0.189059	0.102704
0.088399	0.087056	0.042287
0.216204	0.890369	0.320608
0.389253	0.337803	0.215533
0.032561	0.113636	0.078268
0.075977	0.105489	0.115978
0.482991	0.569244	0.181052
0.021357	0.107461	0.07079
0.098699	0.085357	0.061864
0.072518	0.107405	0.07231
0.013122	0.280152	0.266787
0.036124	0.182746	0.145006
0.123471	0.055616	0.115233
0.117415	0.063216	0.154173
0.194865	0.222321	0.217999
0.062205	0.162751	0.150304
0.100849	0.134389	0.199143
0.081834	0.195793	0.223595
0.109879	0.49503	0.03489
0.258296	0.442514	0.649228

igf1	1.075681	0.568947	0.793716	0.054185		0.302491	0.169942	0.618778
igf1r	-0.058199	-0.089306	-0.001003	-0.109374		0.085374	0.200164	0.116056

	p-value			
JFLS-Ch		CFLS v JFLS	CFLS v CFLS-Ch	JFLS v JFLS-Ch
0.148332		0.358801806	0.498953996	0.148631537
0.170262		0.143737124	0.051195666	0.194188615
0.261087		0.302147689	0.036995654	0.284270102
0.215764		0.035378721	0.32459766	0.037520012
0.136839		0.191607216	0.349619412	0.076759204
0.247285		0.347701293	0.303753437	0.062685908
0.174597		0.355464648	0.174486835	0.331796731
0.26299		0.009373251	0.223968537	0.068593221
0.058087		0.437771831	0.440871092	0.084419054
0.099112		0.336743393	0.237879729	0.485131257
0.110946		0.360581881	0.204060269	0.22341652
0.29298		0.031979944	0.034243018	0.228512416
0.204818		0.048926834	0.224789925	0.174161286
0.05586		0.393097146	0.050551613	0.073064373
0.318613		0.217354011	0.270755384	0.241182918
0.314635		0.466672202	0.182330216	0.48803451
0.063455		0.006166805	0.088144014	0.341276685
0.027315		0.21539281	0.485418896	0.023475028
0.283736		0.163431612	0.418891163	0.473275297
0.091357		0.473042889	0.493381895	0.067419641
0.203331		0.422900413	0.410278436	0.490411806
0.108663		0.056429885	0.243269909	0.062618012
0.491097		0.227108238	0.43383064	0.155599435
0.372674		0.319477428	0.199464299	0.185989664
0.256918		0.479698467	0.316955159	0.477290488
0.223419		0.332575884	0.030756725	0.042253961
0.128753		0.395332601	0.036246658	0.128509708
0.277051		0.212975955	0.270983868	0.325513711
0.092898		0.443147365	0.30801025	0.048721995
0.030748		0.115039175	0.048374178	0.050171174
0.232249		0.048658277	0.382191464	0.173546361
0.348168		0.177603238	0.138927269	0.134178461
0.113317		0.156790552	0.067272455	0.239358937
0.019586		0.460922734	0.234484099	0.024102592
0.110524		0.292241659	0.386566676	0.343252916
0.085427		0.099720308	0.383886199	0.252345923
0.054269		0.388714424	0.288476578	0.349603736
0.111395		0.054979239	0.122654916	0.067793869
0.035661		0.089926797	0.019510263	0.260432211
0.097374		0.286739367	0.256443894	0.208880098
0.191172		0.287705831	0.050403916	0.467888275
0.02572		0.404951658	0.326141052	0.361341605
0.09559		0.342010947	0.099231309	0.193040218
0.304388		0.230642271	0.473037746	0.485114133
0.450504		0.050148593	0.027046389	0.496700983

0.485499	0.258711428	0.032344283	0.089365056
0.037879	0.264751325	0.408321311	0.099490604
